# Engineered ATP-Loaded Extracellular Vesicles Derived from Mesenchymal Stromal Cells: A Novel Strategy to Counteract Cell ATP Depletion in an In Vitro Model

**DOI:** 10.3390/ijms26073424

**Published:** 2025-04-05

**Authors:** Maria Antonietta Grignano, Silvia Pisani, Marilena Gregorini, Giorgia Rainaudo, Maria Antonietta Avanzini, Stefania Croce, Chiara Valsecchi, Gabriele Ceccarelli, Tefik Islami, Elisabetta Margiotta, Valentina Portalupi, Andreana De Mauri, Emma Diletta Stea, Eleonora Francesca Pattonieri, Paolo Iadarola, Simona Viglio, Bice Conti, Teresa Rampino

**Affiliations:** 1 Unit of Nephrology, Dialysis and Transplantation, Fondazione IRCCS Policlinico San Matteo, 27100 Pavia, Italy; ma.grignano@smatteo.pv.it (M.A.G.); m.gregorini@smatteo.pv.it (M.G.); giorgia.rainaudo01@universitadipavia.it (G.R.); t_islami@hotmail.com (T.I.); el.margiotta@smatteo.pv.it (E.M.); v.portalupi@smatteo.pv.it (V.P.); a.demauri@smatteo.pv.it (A.D.M.); e.stea@smatteo.pv.it (E.D.S.); e.pattonieri@smatteo.pv.it (E.F.P.); t.rampino@smatteo.pv.it (T.R.); 2 Department of Drug Sciences, University of Pavia, Viale Torquato Taramelli 12, 27100 Pavia, Italy; bice.conti@unipv.it; 3 Department of Internal Medicine and Therapeutics, University of Pavia, 27100 Pavia, Italy; 4 Pediatric Haematology/Oncology, Fondazione IRCCS Policlinico San Matteo, 27100 Pavia, Italy; ma.avanzini@smatteo.pv.it (M.A.A.); c.valsecchi@smatteo.pv.it (C.V.); 5 Cell Factory and Center for Advanced Therapies, Fondazione IRCCS Policlinico San Matteo, 27100 Pavia, Italy; s.croce@smatteo.pv.it; 6 Human Anatomy Unit, Department of Public Health, Experimental Medicine and Forensic, University of Pavia, 27100 Pavia, Italy; gabriele.ceccarelli@unipv.it; 7 Centre for Health Technologies (CHT), University of Pavia, 27100 Pavia, Italy; 8 Department of Biology and Biotechnologies “L. Spallanzani”, University of Pavia, 27100 Pavia, Italy; paolo.iadarola@unipv.it; 9 Lung Transplantation Unit, IRCCS Policlinico San Matteo Foundation, 27100 Pavia, Italy; simona.viglio@unipv.it; 10 Department of Molecular Medicine, University of Pavia, 27100 Pavia, Italy

**Keywords:** engineered extracellular vesicles, renal ischemia injury, ATP delivery system, mesenchymal stromal cells, renal transplant

## Abstract

The use of adenosine triphosphate (ATP) has shown promising effects in alleviating ischemic damage across various tissues. However, the penetration of ATP into kidney tubular cells presents a challenge due to their unique anatomical and physiological properties. In this study, we introduce a novel bioinspired drug delivery system utilizing extracellular vesicles (EVs) derived from mesenchymal stromal cells (MSCs) and engineered to carry ATP. ATP-loaded liposomes (ATP-LPs) and ATP-loaded EVs (ATP-EVs) were prepared using microfluidic technology, followed by characterization of their morphology (DLS, NTA, SEM, TEM), ATP content, and release rate at 37 °C (pH 7.4). Additionally, the delivery efficacy of ATP-LPs and ATP-EVs was evaluated in vitro on renal cells (HK2 cells) under chemically induced ischemia. The results indicated successful ATP enrichment in EVs, with ATP-EVs showing no significant changes in morphology or size compared to naïve EVs. Notably, ATP-EVs demonstrated superior ATP retention compared to ATP-LPs, protecting the ATP from degradation in the extracellular environment. In an ATP-depleted HK2 cell model, only ATP-EVs effectively restored ATP levels, preserving cell viability and reducing apoptotic gene expression (BCL2-BAX). This study is the first to successfully demonstrate the direct delivery of ATP into renal tubular cells in vitro using EVs as carriers.

## 1. Introduction

Oxygen deprivation during ischemia leads to cellular dysfunction, which can progress to irreversible damage or necrosis if the cell’s capacity to switch to anaerobic metabolism is overwhelmed. In this context, the primary cellular event is the depletion of energy substrates, caused by changes in mitochondrial volume and structure, which can persist even after reperfusion [1,2,3,4]. Kidney tubular cells are especially vulnerable to ischemia, experiencing rapid damage, including mitochondrial disruption, alterations in mitochondrial cristae, increased permeability of transition pores, cytochrome-C translocation, and activation of apoptosis pathways involving Bcl-2 and caspase-3 [1,2,3,4]. As a result, improving cell resilience to ischemia and preventing adenosine triphosphate (ATP) depletion is crucial for enhancing tissue quality. Currently, marginal ischemic organs used for kidney transplantation are perfused ex vivo at 4 °C in a machine perfusion system with a solution that provides energy substrates, such as adenosine, to support cellular metabolism and preserve structural integrity [5,6]. However, administering exogenous ATP during hypothermic machine perfusion (HMP) is not an effective strategy for replenishing ATP reserves in tissues. This is due to the rapid hydrolysis of ATP, which is further accelerated in vivo by ectoenzymes, as well as its poor cellular penetration resulting from its hydrophilic nature [7,8].

In recent years, there has been increasing interest in using mesenchymal stromal cells (MSCs) as a promising alternative for enhancing tissue recovery. MSCs have demonstrated the ability to promote tissue regeneration, suppress inflammation, and modulate the immune response [9,10,11,12,13,14,15,16,17]. Notably, MSCs can transfer mitochondria and release bioactive factors that support ATP production and cellular metabolism, potentially overcoming the limitations of direct ATP administration.

However, their clinical application is often hindered by a slow onset of action or lack of efficacy, which may be attributed to poor bioavailability and off-target effects following systemic administration. In addition, various kinds of MSC sources (bone marrow, adipose tissue, cord blood, etc.) may act differentially, leading to even pro-tumorigenic effects in some conditions [18,19,20,21,22,23].

MSCs can exert their therapeutic properties through either cell–cell contacts or paracrine effects [22,24].

Paracrine action, in particular the release of extracellular vesicles (EVs), has been reported as the fundamental mode of action for MSCs.

EVs influence various cellular processes, including proliferation, apoptosis, and transcriptional regulation, through their content of miRNA, mRNA, lipids, and proteins. Various preclinical models of acute kidney injury (AKI) and chronic kidney disease (CKD) have shown that MSC-derived EVs can effectively improve kidney damage, highlighting their potential as a treatment for kidney failure [25].

Due to their unique properties, EVs offer several advantages over MSCs, such as the ability to cross biological barriers, enhanced stability, biocompatibility, low immunogenicity, and low toxicity. Importantly, EVs are unlikely to cause tumor formation, making them a safer alternative. In fact, their potential in this regard is increasingly recognized, and they are being explored as promising nanocarriers for chemotherapy, providing a novel therapeutic approach [26,27,28,29,30,31,32,33].

As a result, the use of EVs as drug delivery systems has gained considerable attention in recent years. Both naïve and engineered EVs have been extensively explored as carriers for a wide range of therapeutic cargoes, including small molecules, proteins, miRNAs, and siRNAs [34,35,36,37,38,39]. In a previous study using a rat donation after circulatory death model, we demonstrated that MSCs and EVs help to preserve the renal tissue structure during ischemia by upregulating NADH-ubiquinone oxidoreductase, which promotes ATP synthesis [40]. Additionally, EVs supply ATP and adenosine to the tissue by enhancing CD73-adenosinergic signaling, thereby facilitating kidney repair [40,41].

Similarly to EVs, liposomes (LPs) are well-suited for use as delivery vehicles due to their phospholipid bilayer structure. Furthermore, liposomes have demonstrated effectiveness in reducing ischemic damage across various tissues [42,43,44,45,46,47,48,49,50,51,52,53,54,55,56,57,58].

Unfortunately, liposomes generally lack specific targeting properties aimed at the kidneys. Moreover, delivering drugs efficiently and safely to renal tubular cells is challenging due to their anatomical and physiological properties, such as tight junctions, which hinder liposome penetration [59,60].

In contrast, EVs offer distinct advantages over liposomes as delivery vectors. Their negatively charged surfaces provide greater permeability and stability, while their surface molecules enhance targeting capabilities [61,62].

Considering these factors, we hypothesize that increasing ATP concentration within EVs could be an effective approach to restore ATP levels during renal ischemia, with the goal of promoting renal tubular cell regeneration. While the hydrophilic nature of ATP limits its ability to cross the cell membrane, this characteristic may actually aid in its encapsulation within EVs. In this study, we use a microfluidic technique to engineer EVs loaded with ATP and assess their potential to alleviate ATP depletion in renal tubular cells. Additionally, we compare the effectiveness of ATP-loaded EVs with that of ATP-loaded liposomes to identify the most efficient delivery system for boosting ATP levels and supporting cell viability.

## 2. Results

### 2.1. MSC Characterization

**Figure 1:**
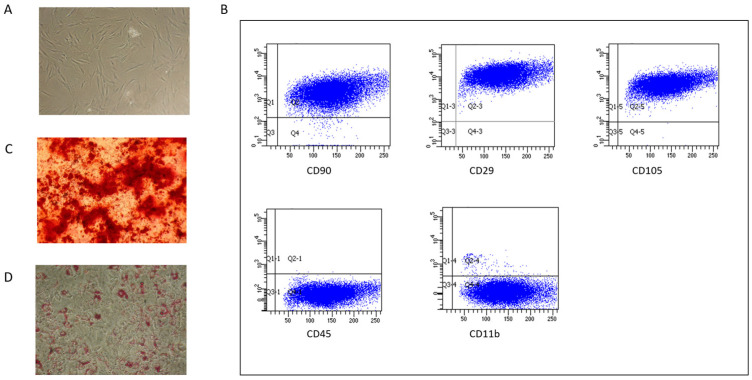
Pig BM-MSC characterization. (**A**) Spindle shape morphology. (**B**) Surface marker expression by flow-cytometry. (**C**) Osteogenic differentiation evidenced by calcium deposition stained by Alizarin red. (**D**) Adipogenic differentiation evidenced by lipid droplets stained with Oil Red.

Pig bone marrow MSCs (BM-MSCs) were characterized based on the minimal criteria established for human MSCs [63]. They exhibited plastic adherence and a fibroblast-like morphology. Surface expression analysis showed they were positive for CD90, CD29, and CD105, and negative for CD45 and CD11b. Additionally, pig BM-MSCs demonstrated the ability to differentiate into the osteogenic lineage in vitro, as indicated by calcium deposition, and into the adipogenic lineage, as evidenced by the presence of fat droplets [64] (Figure 1).

### 2.2. Liposomes and EVs Characterization

#### 2.2.1. Dimensional Characterization

**Figure 2:**
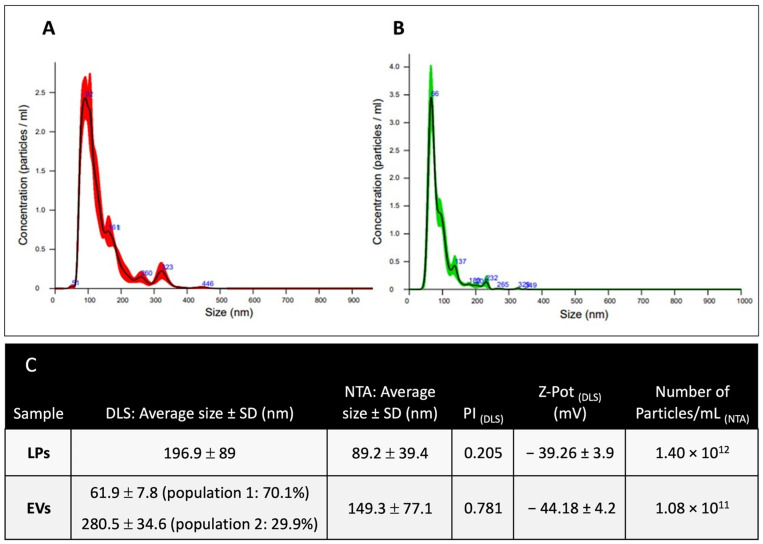
NTA of EVs (Panel (**A**)) and liposomes (Panel (**B**)) following the isolation/preparation. Data are expressed as the concentration of particles per ml of medium (ordinate axis) or as the size of particles in nanometers (abscissa axis). (**C**) Table reporting data about dimension, electrical potential, and concentration of LPs and EVs.

Dimensional characterization of placebo LPs and naïve EVs was performed using both nanotracking analysis (NTA) and dynamic light scattering (DLS). The data presented in Figure 2 compare the physicochemical properties of liposomes (LPs) and extracellular vesicles (EVs) in terms of size distribution, polydispersity, zeta potential, and particle concentration. DLS results show that LPs have an average size of 196.9 ± 89 nm, indicating a relatively uniform population. In contrast, EVs display a bimodal size distribution, consisting of a smaller population (61.9 ± 7.8 nm, 70.1%) and a larger one (280.5 ± 34.6 nm, 29.9%). This heterogeneity is reflected in the higher polydispersity index (PI) of EVs (0.78) compared to LPs (0.21), suggesting greater variability in EV size. NTA measurements support the DLS findings, showing that LPs have a smaller average size (89.2 ± 39.4 nm) compared to EVs (149.3 ± 77.1 nm). The discrepancy between DLS and NTA values is expected as DLS measures the hydrodynamic diameter based on scattering intensity changes from a bulk sample, whilst NTA directly measures individual particle diffusion. The zeta potential (Z-pot) values indicate that both LPs (−39.26 mV) and EVs (−44.18 mV) have highly negative surface charges, contributing to their colloidal stability. LP samples have a significantly higher particle concentration (1.40 × 10^12^ particles/mL) compared to EVs (1.08 × 10^11^ particles/mL), likely due to differences in their production methods. The controlled microfluidic synthesis of LPs tends to yield a higher number of smaller, uniform particles, while EVs, derived from biological sources, exhibit a broader size range and lower particle yield.

**Figure 3:**
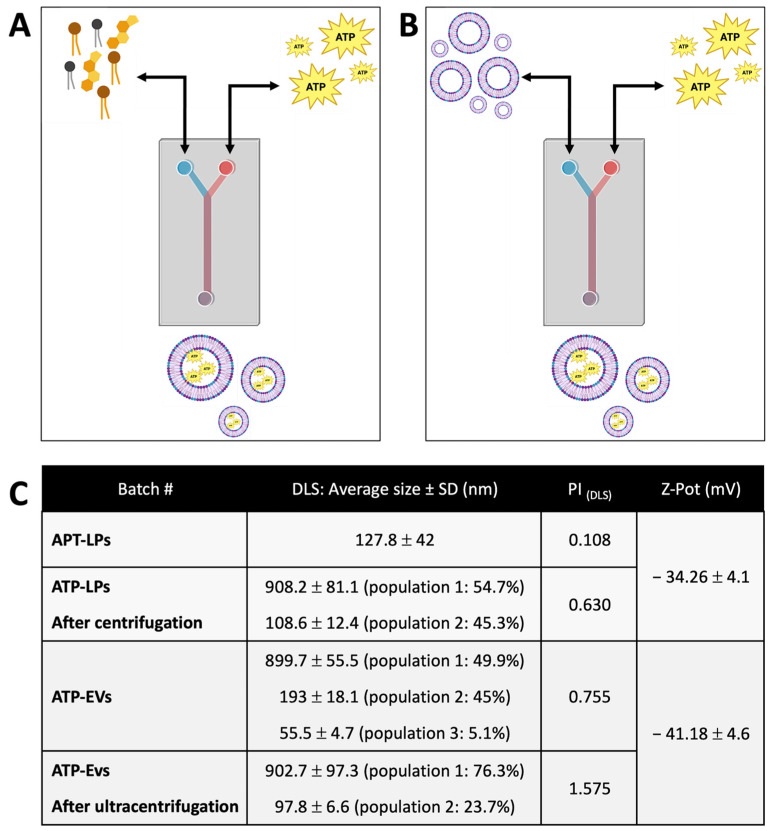
(**A**) Schematic representation of direct method for ATP-LPs loading through microfluidic technique; (**B**) schematic representation of indirect method for ATP-LPs and ATP-EVs loading through microfluidic technique; (**C**) results of dimensional characterization (nm ± SD and polydispersity index) and electrical potential using DLS.

The size distribution and polydispersity index (PI) of ATP-LPs and ATP-EVs, obtained through DLS analysis, are shown in Figure 3. ATP-LPs and ATP-EVs were prepared using different loading methods (direct vs. indirect) and purified via centrifugation or ultracentrifugation. The direct loading method resulted in slightly smaller ATP-LPs (127.8 ± 42 nm, PI = 0.11) compared to the indirect method (142.9 ± 31 nm, PI = 0.20). The lower PI of ATP-LPs from the direct method suggests a more uniform size distribution, reflecting a more efficient and controlled loading process. ATP-EVs exhibited a heterogeneous size distribution, with three distinct populations: 899.7 ± 55.5 nm (49.9%), 193 ± 18.1 nm (45%), and 55.5 ± 4.7 nm (5.1%). The ATP-EVs also showed a significant increase in particle size, particularly with the larger fraction, which was absent in naïve EVs (Figure 3). This suggests that ATP loading via the indirect method may induce aggregation or vesicle fusion, leading to increased size variability. The high PI (0.75) of ATP-EVs, similar to that of naïve EVs (0.78), indicates considerable size variability, likely due to the natural heterogeneity of EVs.

After centrifugation, ATP-LPs experienced a significant increase in size, displaying a bimodal distribution (from 908.2 ± 81.1 nm to 108.6 ± 12.4 nm), indicating the formation of larger aggregates or non-encapsulated components. The final PI (0.63) remained relatively high, suggesting some residual heterogeneity.

Following ultracentrifugation, ATP-EVs retained two distinct populations, with a larger fraction (~900 nm) and a smaller fraction (~97 nm), indicating that the process mainly concentrated the vesicles without significantly reducing size heterogeneity. The consistently high polydispersity index (PI = 1.57) further supports the idea that ultracentrifugation primarily affects vesicle concentration rather than selectively refining the size distribution. The Z-pot of LPs (−34.26 ± 4.1 mV) and EVs (−41.18 ± 4.6 mV) after purification shows that they maintain highly negative surface charges.

#### 2.2.2. Morphometric Analysis

**Figure 4:**
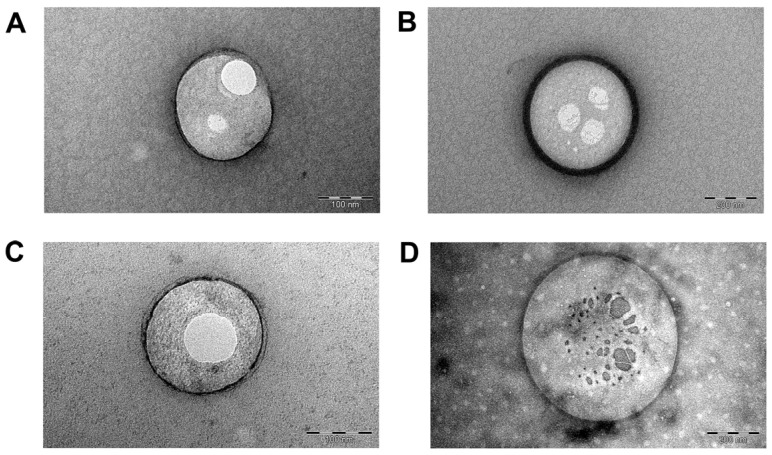
TEM images of naïve small (**A**) and large (**B**) EVs derived from porcine bone marrow MSCs, as well as small (**C**) and large (**D**) EVs after ATP loading and ultracentrifugation (50K, 150K, and 300K magnification).

The morphology and size of LPs and EVs were analyzed using TEM, with the images presented in Figure 4. TEM images confirmed the predominantly spherical shape of both liposomes and EVs, consistent with the results from DLS and NTA analyses. Cryo-EM was also performed to examine the 3D morphology of naïve EVs, further confirming their round shape. The average bilayer thickness of EVs was measured to be 28.99 ± 14.98 nm (Figure S1).

TEM analysis was also conducted to assess the morphology of vesicles after ATP loading and subsequent ultracentrifugation (Figure 4C and Figure S2). ATP-EVs displayed a more irregular shape and larger size compared to the spherical morphology observed in Figure 4B before the indirect loading process through the microfluidic channels.

### 2.3. ATP Quantification in Liposomes and EVs

**Figure 5:**
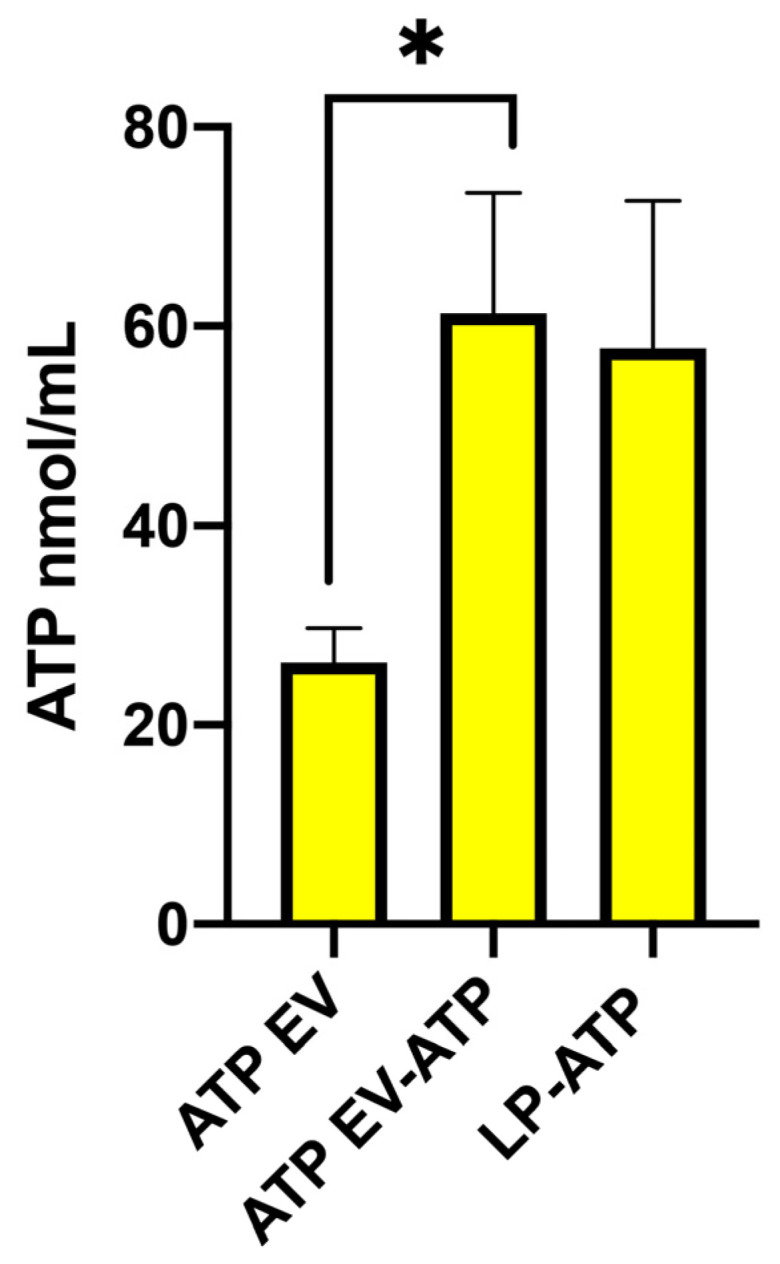
ATP level in naïve EVs (EVs), ATP-loaded EVs (ATP-EVs), and ATP-loaded liposomes. Data are expressed as mean and standard deviations. * *p* < 0.05.

Naïve extracellular vesicles (EVs) had a baseline ATP concentration of 26.22 ± 3.45 nm/mL. Indirect loading of ATP into EVs via the microfluidic technique significantly boosted ATP levels, nearly doubling their concentration to 54.88 ± 16.23 nm/mL (*p* < 0.01). Direct encapsulation of ATP within liposomes resulted in ATP concentrations similar to those in microfluidically loaded EVs. In contrast, indirect encapsulation in liposomes led to a lower ATP concentration (30.98 ± 8.47 nm/mL) compared to the direct method (57.84 ± 14.80 nm/mL, *p* < 0.05) (Figure S3). Due to its higher loading efficiency, the direct liposomal encapsulation method was chosen for subsequent ATP loading experiments as it showed comparable efficacy to ATP-loaded EVs (*p* < 0.05) (Figure 5).

### 2.4. ATP In Vitro Release Study

**Figure 6:**
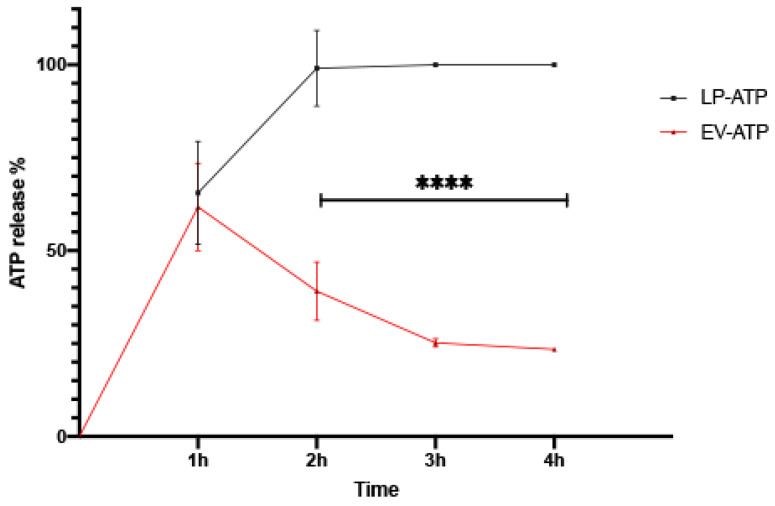
In vitro release of ATP from liposome batches obtained by microfluidics (black line, ATP-LPs) and from extracellular vesicles isolated from mesenchymal stromal cells (red line, ATP-EVs) at 37 °C in PBS (pH = 7.4). **** *p* > 0.00001.

To evaluate ATP retention, the release profiles of ATP-LPs and ATP-EVs were assessed at 37 °C (Figure 6). In the first hour, both formulations showed a similar release pattern, with ATP-LPs releasing 65.52% ± 13.84% and ATP-EVs releasing 61.76% ± 11.76% (Mean ± SD). However, notable differences appeared in their release kinetics over time. ATP was completely released from liposomes within two hours, while the EVs stopped releasing ATP after the first hour, retaining about 39% of the encapsulated ATP after four hours (ATP-LPs vs. ATP-EVs, *p* < 0.00001). This discrepancy emphasizes the distinct release mechanisms of the two systems.

### 2.5. Cell ATP Concentration and Viability in Basal Conditions and After Chemical Ischemia

**Figure 7:**
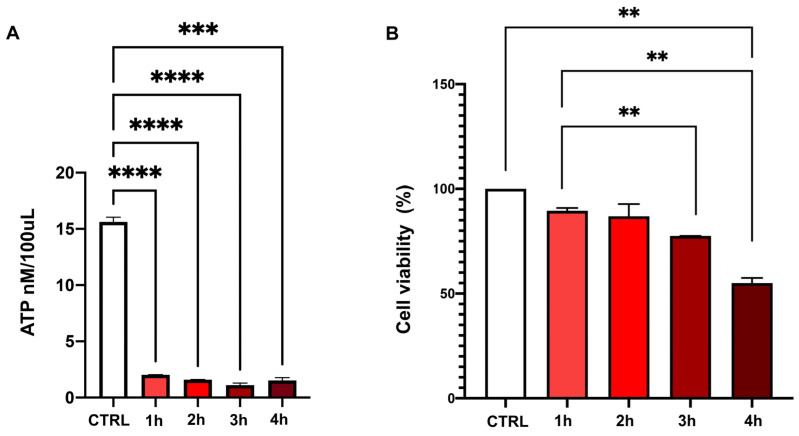
(**A**) Intracellular ATP concentration in HK2 after 1, 2, 3, or 4 h of chemical ischemia injury. Columns representing the intracellular ATP levels expressed as mean and standard deviations. *** *p* < 0.005; **** *p* < 0.001. (**B**) HK2 viability after 1, 2, 3, or 4 h of ATP depletion expressed as median with IQR. ** *p* < 0.01.

To simulate ischemic damage in vitro, ATP depletion was induced in HK2 (human kidney 2) cells by varying treatment durations. All treatment times (1 h, 2 h, 3 h, and 4 h) resulted in a significant decrease in ATP levels compared to the control group (CTRL; complete medium) (mean ± SD: CTRL 15.62 ± 0.42 vs. 1 h 2.02 ± 0.03, 2 h 1.59 ± 0.01, 3 h 1.11 ± 0.18, *p* < 0.0001; 4 h 1.52 ± 0.26, *p* < 0.005) (Figure 7A). However, cell viability significantly decreased after 3 h of treatment compared to 1 h (median (25th–75th percentile): 1 h 89.47 (87.50–90.90) vs. 3 h 77.50 (77.48–77.52), *p* < 0.01; 4 h 55.00 (55.00–57.50), *p* < 0.01) (Figure 7B). Based on these results, a two-hour treatment duration was selected for subsequent HK2 cell conditioning experiments.

### 2.6. ATP Loading into HK2 Cells

**Figure 8:**
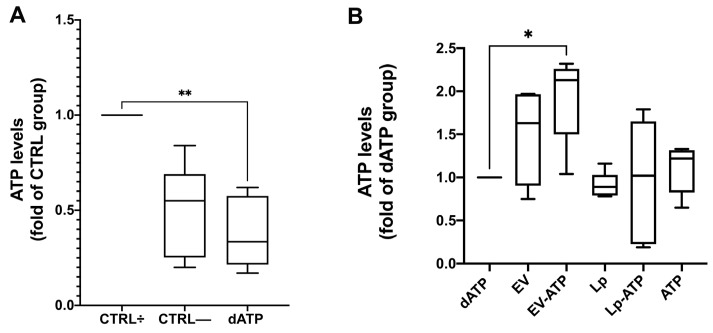
Enzyme-linked immunosorbent assay. Box-plots representing the intracellular ATP levels expressed as median and 2.5–97.5 percentile and as fold change to CTRL+, ** *p* < 0.01. (Panel (**A**)) or as fold change of dATP * *p* < 0.05 (Panel (**B**)). CTRL+: cells cultured in complete medium; CTRL-: starved cells; dATP: ATP-depleted cells after four hours of incubation in DMEM-LG; EV: ATP-depleted cells after four hours of incubation in DMEM-LG with naïve EVs; EV-ATP: ATP-depleted cells after four hours of incubation in DMEM-LG with ATP-EVs; Lp: ATP-depleted cells after four hours of incubation in DMEM-LG with empty liposomes; LP-ATP: ATP-depleted cells after four hours of incubation in DMEM-LG with ATP-LPs; ATP: ATP-depleted cells after four hours of incubation in DMEM-LG with free ATP.

After two hours of ATP depletion followed by four hours in DMEM-LG (dATP), HK2 cells showed significantly reduced ATP levels compared to the control group (CTRL+) (median (25th–75th percentile): dATP 0.33 (0.21–0.57) vs. CTRL; *p* < 0.01) (Figure 8A). Both naïve and ATP-loaded extracellular vesicles (EVs) effectively counteracted the ATP depletion, but treatment with ATP-EVs showed the most significant recovery (median (25th–75th percentile): EV-ATP 2.13 (1.5–2.26) vs. dATP; *p* < 0.05). In contrast, ATP-LPs did not enhance intracellular ATP levels, which remained similar to those in the dATP, empty liposomes (placebo), and free-ATP treatments (Figure 8B).

### 2.7. HK2 Viability

**Figure 9:**
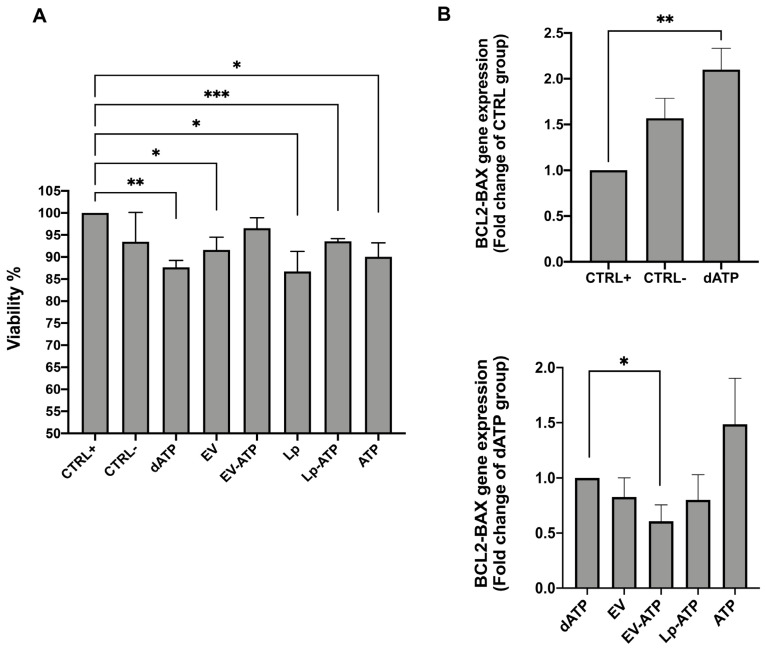
Cell vitality. (**A**) Trypan blue dye. Columns representing the cell vitality expressed as mean and standard deviations. * *p* < 0.05; ** *p* < 0.01; *** *p* < 0.005. (**B**) BCL2-BAX mRNA expression after ATP depletion and conditioning treatment expressed as fold change in CTRL + (top) and in dATP (bottom). * *p* < 0.05; ** *p*< 0.01. CTRL+: cells cultured in complete medium; CTRL-: starved cells; dATP: ATP-depleted cells after four hours of incubation in DMEM-LG; EV: ATP-depleted cells after four hours of incubation in DMEM-LG with naïve EVs; EV-ATP: ATP-depleted cells after four hours of incubation in DMEM-LG with ATP-EVs; Lp: ATP-depleted cells after four hours of incubation in DMEM-LG with empty liposomes; LP-ATP: ATP-depleted cells after four hours of incubation in DMEM-LG with ATP-LPs; ATP: ATP-depleted cells after four hours of incubation in DMEM-LG with free ATP.

Compared to the control group, all conditioning treatments except for ATP-EVs resulted in reduced HK2 cell viability, as measured by Trypan blue dye. (mean ± SD: CTRL 100 ± 0.00 vs. dATP 87.68 ± 1.55; *p* < 0.01, vs. EV 91.60 ± 2.91; *p* < 0.05, vs. Lp 86.72 ± 4.55; *p* < 0.05, vs. Lp-ATP 93.58 ± 0.59; *p* < 0.005, vs. ATP 90.04 ± 3.16; *p* < 0.05). ATP-EVs maintained cell viability comparable to both the positive (CTRL+) and negative (CTRL-) control groups (Figure 9A).

This finding was further supported by the apoptotic gene expression results.

Real-time polymerase chain reaction (RT-PCR) revealed an approximately 50% increase in BCL2-BAX mRNA expression in ATP-depleted HK2 cells after four hours of incubation in DMEM-LG, compared to their respective control cells (CTRL+ vs. dATP; *p* < 0.01) (Figure 9B top).

This upregulation was reduced by about 40% in HK2 cells treated with ATP-loaded EVs following ATP depletion injury (dATP vs. EV-ATP; *p* < 0.05). Other conditioning treatments did not significantly affect BCL2-BAX gene expression (Figure 9B bottom).

## 3. Discussion

This study introduces a novel microfluidic approach to engineer mesenchymal stem cell (MSC)-derived extracellular vesicles (EVs) loaded with ATP. Microfluidic technologies have gained significant attention in recent years for efficiently producing lipid nanoparticles and encapsulating small-molecule drugs, thus offering several advantages, including precise control over vesicle size, composition, and drug loading efficiency. Additionally, these methods enable rapid, non-invasive, and scalable production while minimizing external manipulation, thereby preserving EVs’ structural and functional integrity. Leveraging these benefits, this approach has significant potential to enhance the therapeutic efficacy of EV-based drug delivery systems [65,66].

In this study, we evaluate the effects of liposomes, widely used lipid-based carriers for drug and molecule delivery, and EVs, which have shown therapeutic potential in an experimental ischemia/reperfusion model [67,68]. As a cornerstone in nanomedicine, liposomes serve as a benchmark for comparison. While both liposomes and EVs are phospholipid-based structures, EVs offer key advantages due to their biological origin, including enhanced biocompatibility, reduced immunogenicity, and intrinsic cell-targeting properties. By comparing these two nanocarrier systems, we aim to deepen our understandings of their therapeutic potential and applications.

The results highlight the effectiveness of both microfluidic EV loading and direct liposomal encapsulation in achieving high ATP concentrations. Notably, direct liposomal encapsulation showed superior efficiency over indirect methods, making it the preferred approach for further studies. The comparable ATP levels between ATP-EVs and directly loaded liposomes suggest both systems hold promise for therapeutic applications requiring efficient ATP delivery.

However, size distribution analysis reveals key differences between ATP-LPs and ATP-EVs as liposomes show a more uniform size when directly loaded while EVs remain highly heterogeneous due to their biological origin. ATP loading appears to induce vesicle aggregation in EVs, increasing their size variability. Centrifugation and ultracentrifugation further affect particle distribution, with ATP-LPs forming aggregates and ATP-EVs retaining a bimodal size profile. The persistently high PI of ATP-EVs reflects their inherent heterogeneity (e.g., exosomes and microvesicles), suggesting that ultracentrifugation primarily concentrates vesicles rather than refining their size distribution. These findings emphasize the need for further optimization to enhance EV homogeneity for therapeutic applications. Additionally, the higher negative zeta potential of EVs may influence their interactions with biological membranes, potentially affecting their stability and biodistribution profiles compared to LPs [69].

Although EVs hold great potential as drug delivery systems and therapeutic agents, their pharmacokinetic and pharmacodynamic profiles remain incompletely understood [70]. This study highlights key differences in ATP release kinetics between liposomes and EVs. While liposomes released nearly all their ATP within two hours, the ATP release profile from EVs slows after 1 h and remains incomplete.

The hindering of ATP release by EVs may be attributed to both the biological nature of the membrane and to the unique release mechanisms and potential reuptake processes.

Unlike liposomes, which primarily release their contents through passive diffusion, EVs are likely to use more regulated pathways that involve specific membrane proteins or signaling cascades to control ATP release.

This could result in an initial burst of ATP followed by a reduction or cessation of release over time. Additionally, EVs might have the ability to reabsorb or re-incorporate ATP from their surroundings, leading to a decrease in measurable extracellular ATP levels as incubation progresses.

Understanding these dynamic processes is essential for accurately interpreting the ATP release profiles from EVs and requires further research into the molecular mechanisms that govern their cargo release and uptake [71,72].

Moreover, EVs exhibit a superior ability to cross biological barriers and deliver ATP intracellularly. Their ATP retention mechanism seems to be crucial for targeted delivery to ischemic tissues, where ATP’s hydrophilic nature and rapid hydrolysis by ectonucleotidases limit its passive diffusion and stability. However, further studies on the ATP metabolism within EVs, the enzymatic activity, and the membrane transport mechanisms are needed to fully understand their role in ATP regulation and therapeutic potential [7,8,67].

To investigate the ability of EVs and liposomes to transfer ATP to HK2 cells in an in vitro ischemia model, we first identified the optimal time rank during which ATP levels could decline without compromising cell viability. Our results demonstrated that the combined use of 2-deoxyglucose, a glycolysis inhibitor, and antimycin A, an inhibitor of the mitochondrial respiratory chain, effectively depleted ATP levels within two hours while maintaining cellular survival [73,74].

Following ischemic insult, cells were treated for 4 h with various interventions. Our results showed that ATP-EVs not only successfully transferred their ATP content into the cells but also enhanced cell vitality and reduced apoptosis more effectively than naïve EVs. These findings are consistent with those of Lindoso et al., who demonstrated that naïve vesicles counteract ATP decline in an in vitro renal-cell-ischemia model, protecting against cell death and maintaining transepithelial resistance [75].

The differences in efficacy and effects between LP-ATP and EV-ATP should be explained by their different kinetic delivery methods. LP-ATP releases ATP rapidly into the extracellular environment, leading to its swift degradation and limiting its availability for cellular uptake. As a result, cells exposed to LP-ATP showed reduced viability and increased apoptosis, highlighting its inefficiency in sustaining ATP delivery.

In contrast, EV-ATP ensures a more controlled and prolonged release, enhancing ATP stability and retention at the target site. This sustained availability supports long-term cellular energy balance and signaling, making EVs more effective carriers.

Our findings support the hypothesis that sufficient intracellular ATP levels delivered by EVs protect cells from energy failure caused by ischemia injury. While the use of mesenchymal stromal cell-derived vesicles to address ischemic injury is not new, ATP-engineered EVs as an intracellular energy delivery system represent a significant advancement.

In our model, the use of microfluidic technology improved the encapsulation efficiency while preserving EV integrity and bioactivity.

Microfluidics offers several advantages, including higher reproducibility, scalability, and reduced processing times compared to conventional loading techniques. By optimizing this engineering strategy, we aimed to maximize ATP retention and improve the therapeutic potential of EVs for targeted delivery.

Recent literature underscores the importance of establishing standardized data reporting frameworks to enhance comparability and reproducibility in EV research. For instance, Piffoux et al. propose a comprehensive data reporting framework to support standardization in EV engineering and loading for clinical applications Additionally, Ma et al. discuss various bioengineering strategies for EVs, emphasizing the need for robust biomanufacturing processes and regulatory considerations to facilitate clinical translation [76,77].

However, this study has some limitations. First, the use of an in vivo model is necessary to validate our findings. Second, further experiments are required to assess the impact of ATP-loaded EVs on cellular energy metabolism.

To our knowledge, this is the first report of vesicles directly delivering ATP to epithelial tubular cells.

The clinical translation of engineered EVs holds significant promise. By leveraging their innate biocompatibility and capacity for cargo delivery, EVs can be tailored for targeted therapy in various diseases.

If successful in both acute and chronic injuries, this approach could benefit millions of patients, not only in transplantation but also in various ischemic-related conditions, such as cardiovascular and vascular diseases.

However, challenges such as large-scale production, standardization, and ensuring safety and efficacy must be addressed.

The study is the first to demonstrate that EVs can be effectively loaded with ATP and successfully deliver it. Moreover, ATP-loaded EVs were shown to counteract ATP depletion in HK2 cells, a well-known in vitro model of ischemia. In conclusion, this study lays the foundation for future research aimed at improving and preserving organ structure by providing ATP after an ischemic insult, such as post-transplantation.

## 4. Materials and Methods

### 4.1. BM-MSCs Expansion and Characterization

Mesenchymal stromal cells (MSCs) were isolated and expanded from pig bone marrow (BM) as previously described [63]. Briefly, BM aspirates were obtained from the posterior iliac crest of 4 large white, 6-month-old piglets (mean weight 25 ± 5 kg) under general anesthesia with 3% isoflurane. Mononuclear cells (MNCs) were isolated from pig heparinized BM by density gradient centrifugation (Ficoll 1.077 g/mL; Lymphoprep, Nycomed Pharma, Oslo, Norway). MNCs were cultured on uncoated polystyrene culture flasks (Corning Costar, Celbio, Milan, Italy) at a density of 160,000/cm^2^ in low glucose DMEM (DMEM-LG) (Gibco Invitrogen, Paisley, UK) supplemented with gentamicin, 50 μg/mL (Gibco Invitrogen), and 10% fetal calf serum (FCS; Mesenchymal Stem Cell Stimulatory Supplements, StemCell Technologies, Vancouver, BC, Canada). Cells were grown at 37 °C in a humidified atmosphere containing 5% CO_2_, and culture medium was replaced twice per week. At ≥80% confluence, cells were detached by Trypsin-EDTA (Sigma-Aldrich, Milan, Italy), and re-plated at a concentration of 4000 cells/cm2 for expansion until passage 4 (P4).

Porcine MSCs were characterized as previously described [58] for the expression of surface antigens CD45 (LifeSpan Biosciences, Seattle, WA, USA), CD11b (BioLegend, San Diego, CA, USA), CD90 (BD PharMingen, San Diego, CA, USA), CD105, and CD29 (Acris Antibodies, Herford, Germany) by flow cytometry, and for the ability to differentiate in vitro into osteoblasts and adipocytes under specific stimuli [64].

### 4.2. Isolation of MSC-EVs

Extracellular vesicles were isolated from MSC supernatants (EVs) collected after overnight starvation. To remove debris, supernatants were centrifuged at 2000× *g* at +4 °C for 20 min. Subsequently, supernatants were ultracentrifuged at 100,000× *g* (Optima L-90K ultracentrifuge; Beckman Coulter, Brea, CA, USA) for 1 h at 4 °C and the pellets containing EVs were resuspended in PBS and ultracentrifuged for another hour. Pellets were then resuspended in PBS containing 1% DMSO and stored at −80 °C.

### 4.3. Liposome Preparation

Liposomes were prepared using 1,2-distearoyl-sn-glycero-3-phosphocholine (DSPC), cholesterol (Chol), and 1,2-dioleoyl-sn-glycero-3-phospho-L-serine (DOPS) as the anionic lipid, all purchased from Merck (Darmstadt, Germany). The preparation was performed using a microfluidic technique with a NanoAssemblr™ Benchtop apparatus (Precision Nanosystems Inc., Vancouver, BC, Canada), equipped with a staggered herringbone micromixer (SHM) to induce rapid mixing through chaotic advection [58,78]. For the formulation, channel 1 of the NanoAssemblr™ was filled with PBS (pH 7.4), while channel 2 contained a lipid stock solution (10 mM) in ethanol, composed of DSPC, Chol, and DOPS in a molar ratio of 47.5:47.5:5. Liposome synthesis was carried out under optimized conditions, with a total flow rate (TFR) of 8 mL/min and a flow rate ratio (FRR) of 3:1 (aqueous phase/lipid phase), resulting in a total batch volume of 1 mL. The process was conducted at a temperature of 25 ± 3 °C to ensure stability and uniformity [79]. Following synthesis, liposomes were collected by centrifugation (Centrifuge 5417R, Eppendorf SrL, Milan, Italy) at 4 °C and 16,400 rpm for 30 min to remove excess ethanol and unincorporated components, ensuring a purified final formulation.

### 4.4. ATP Loading into Liposomes and EV

Adenosine triphosphate magnesium (ATP-Mg^2+^), purchased from Merck, was used for loading into LPs and EVs. ATP-Mg^2+^ loading into LPs was performed using both direct and indirect methods via a microfluidic technique (Figure 4A,B, respectively), whereas only the indirect method was used for ATP loading into EVs. In the direct loading method, channel 1 of the NanoAssemblr™ instrument was filled with an ATP-Mg^2+^ stock solution in PBS (10 mM), while channel 2 contained the lipid stock solution (DSPC:Chol:DOPS 47.5:47.5:5), as described in Section 4.3. ATP loaded liposomes (ATP-LPs) were obtained using a total flow rate (TFR) of 8 mL/min and a flow rate ratio (FRR) of 3:1, with a total batch volume of 1 mL (25 ± 3 °C). In the indirect loading method, channel 1 of the NanoAssemblr™ instrument was again filled with the ATP-Mg^2+^ stock solution (10 mM in PBS), while channel 2 contained either a placebo batch of centrifuged liposomes (prepared as described in Section 4.3) or a batch of EVs resuspended in PBS. Loading was carried out under the same conditions as the direct method (TFR of 8 mL/min, FRR of 3:1, total batch volume of 1 mL at 25 ± 3 °C).

To remove unloaded ATP from ATP-LPs, the samples were centrifuged using a Centrifuge 5417R (Eppendorf) at 4 °C, 16,400 rpm for 30 min. The supernatant was discarded, while the pellet (containing ATP-LPs) was resuspended in 1 mL of PBS (pH 7.4) with 2% (*v*/*v*) Triton X-100. The ATP-LPs were then processed to disrupt the liposome structure and release ATP by vortexing (Advanced IR Vortex Mixer ZX4, Velp Scientifica, Gelderland, The Netherlands) for 1 min at 1600 rpm, incubation in a thermostatic bath (Memmert, Ghiaroni & C, Schwabach, Germany) at 75 °C for 5 min, and sonication (SONICA ultrasonic cleaner, Soltec, Milan, Italy) for 1 min. The samples were then prepared for ATP quantification, as described in Section 2.5. All analyses were performed in triplicate.

For ATP-loaded EVs (ATP-EVs), unloaded ATP was removed using ultracentrifugation (Beckman Coulter) at 4 °C, 100,000× *g* for 1 h. The supernatant was discarded, and the pellet (containing ATP-EVs) was resuspended in 1 mL of PBS (pH 7.4) with 2% (*v*/*v*) Triton X-100. ATP-EVs then underwent the same treatment as ATP-LPs (vortexing, thermostatic incubation, and sonication) before being processed for ATP quantification (Section 4.5). All analyses were performed in triplicate to ensure accuracy and reproducibility.

### 4.5. ATP Quantification in Liposomes and EVs

ATP levels were measured using an enzyme-linked immunosorbent assay (ab83355, ATP Assay Kit; Abcam, Cambridge, UK) following the manufacturer’s instructions. Before use, ATP converter and developer mix was solubilized in 220 μL of ATP assay buffer. A 1 mM ATP standard solution was prepared by diluting 10 μL of a 10 mM ATP stock solution in 90 μL of PBS, and serial dilutions were made to generate ATP standards at 0, 2, 4, 6, 8, and 10 nmol/well.

An ATP reaction mixture (ARM) was then prepared by mixing 44 μL of ATP assay buffer, 2 μL of ATP probe, 2 μL of ATP converter, and 2 μL of developer mix. For ATP quantification, 50 μL of each standard solution was combined with 50 μL of ARM in a 96-well plate. The plate was incubated for 30 min, protected from light, and placed on a rocking platform for gentle mixing. Absorbance was then measured at 570 nm using a Microplate Photometer HiPo MPP-96 (Biosan, Brescia, Italy). An ATP calibration curve was generated by analyzing the absorbance of ATP standards, with the resulting equation (y = 0.0442x − 0.0131, R^2^ = 0.9972). To account for ATP levels in naïve vesicles, in ATP-EVs and in ATP-LPs, 50 μL of each sample was mixed with 50 μL of ARM. All analyses were performed in triplicate to ensure reproducibility.

### 4.6. ATP In Vitro Release Study

An ATP in vitro release test was performed on ATP-LPs and ATP-EVs pellets that were resuspended in 1 mL of PBS and incubated at 37 °C; the ATP release kinetics were also evaluated. A dialysis membrane system (Spectrum™ Spectra/Por™ 2 RC Dialysis Membrane Tubing, Fisher Scientific, Milan, Italy) was used to separate the LPs or EVs from the released ATP by immersing the samples in 2 mL of PBS. To mimic the in vitro hypoxic conditions used for renal cell testing, ATP release was monitored over 4 h. At predetermined time points (1 h, 2 h, 3 h, and 4 h), 100 μL of the external PBS solution was withdrawn and immediately replaced with an equal volume of fresh PBS to maintain constant conditions. ATP cumulative quantification for each sample was then performed as previously described in Section 4.5. All the analysis was performed in triplicate.

### 4.7. Liposomes and EVs Characterization

LPs and EVs were characterized for their dimensions through nanoparticle tracking analysis (NTA, NanoSight LM10, Alfatest, Rome, Italy) and dynamic light scattering (DLS, Nicomp 380ZLS, Particle Sizing Systems, Qi Technologies, Santa Barbara, CA, USA). Morphology was analyzed by transmission electron microscopy (TEM, JEM-1200EXIII, JEOL, Tokyo, Japan), and naïve EVs were also characterized using cryo-electron microscopy (Cryo-EM transmission electron microscope Glacios, Thermo Fisher Scientific, Waltham, MA, USA).

#### 4.7.1. Nanoparticle Tracking Analysis

NTA was conducted on placebo lipid particles and naïve extracellular vesicles to assess their size distribution and concentration. The samples were carefully diluted at a 1:500 ratio using sterile water for injection USP to ensure optimal particle detection and accurate measurements. The analyses were carried out at a controlled temperature of 25 °C to maintain sample stability and consistency.

#### 4.7.2. Dynamic Light Scattering

For DLS analyses, 300 μL of sample containing naïve EVs and 300 μL of sample containing placebo LPs were tested. DLS analysis was also performed on ATP-EVs and ATP-LPs. The parameters set up were the use of channel 10 and an intensity of 100 KHz. The same instrument was used for Z-potential determination of EVs and LPs. A total of 100 µL of EVs or LPs were placed in 2 mL of NaCl solution (0.01 M) pH 7.4. Analyses were performed at 10 mV electric field intensity and at room temperature (25 ± 2 °C).

#### 4.7.3. Transmission Electron Microscopy

Placebo LPs and naïve EVs were analyzed with TEM equipped with the CCD camera Mega View III (JEOL). Samples were previously negatively stained with 2% uranyl acetate to enhance the image contrast. Afterwards, they were placed on the TEM grids made of copper, with a mesh size of 3 mm, and a layer of formvar coated with a thin film of carbon. The samples were incorporated into an amorphous film on the carbon grid, created when the phosphorus salt that surrounded them dried out. Sample preparation and analysis were conducted at 25 ± 2 °C. Sample images were acquired at magnifications of 50 K, 150 K, 300 K.

#### 4.7.4. Cryo-Electron Microscopy

A sample solution containing naïve EVs was put onto cryo-grids for vitrification before being transferred to an electron microscope for image collection. A lacey carbon TH Cu 200 grid (3 mm of diameter) was used for the analysis of the EVs. The grid was also positively charged through the deposition on its surface of 4 μL of magnesium acetate (5 Mm, Sigma-Aldrich) followed by 1 min incubation and 5 min of drying. The sample containing the EVs was first diluted 1:10 in PBS to have a concentration of the starting suspension of 1011 particles/mL; 4 μL of this suspension were deposited on each grid for vitrification, which was then incubated for a few seconds to encourage particle deposition. The vitrification of the EVs was carried out in the Vitrobot System (Thermo Fisher Scientific, Waltham, MA, USA). The grid was then blotted to remove any surplus solution and plunge-frozen in liquid ethane that had been cooled by liquid nitrogen. The temperature at which ethane becomes liquid is only a little bit greater than that at which liquid nitrogen becomes liquid, despite having a substantially higher heat capacity. Since liquid ethane has a melting point of −188 °C, it is sufficiently cold to freeze water, avoiding its crystallization while not boiling off. The cryo-plunging was carried out at a temperature of 4 °C and a relative humidity of 95%. The grids were clipped from the bottom with a metallic ring and from the top with a metallic c-clip. Then, they were placed in the loading station for the visualization of the samples with the electronic microscope. Cryo-EM images were processed using ImageJ software 1.54p (17 February 2025-NIH) in order to evaluate the EVs’ bilayer thickness. Three measurements were made for each EV visible in the image. Data were then re-elaborated by calculating the arithmetic mean of the measurements and the standard deviation.

### 4.8. Renal Tubular Cell Culture

HK2 (Merck) is a proximal tubular epithelial cell line derived from a normal kidney. Cells were grown in DMEM-LG/F12 medium (Euroclone SpA, Milan, Italy) supplemented with 8% [*v*/*v*] FCS (R&D Systems, Minneapolis, MN, USA), 100 U/mL penicillin-streptomycin (Merck), 5 μg/mL insulin, 5 μg/mL transferrin, and 5 ng/mL sodium selenite (Gibco-Thermo Fisher Scientific, Waltham, MA, USA). Cells were grown at 37 °C in a humidified 5% CO_2_ condition. During the conditioning interventions, the cells were cultured with DMEM-LG in the absence of FCS.

### 4.9. Cell ATP Depletion Injury Model

To induce an injury that closely mimics key aspects of renal tubule damage during renal ischemia, 60–70% confluent HK2 cells were incubated in PBS, in the presence of 10 mM 2-deoxyglucose (Merck, Rahway, NJ, USA) to inhibit glycolysis, and 1 μM antimycin A (Merck) to block the mitochondrial respiratory chain at complex III. This combination of inhibitors prevents any substrate oxidation and leads to the nearly complete depletion of ATP stores. Preliminary set up experiments involved incubating HK2 cells in ATP depletion medium for 1, 2, 3, or 4 h at 37 °C and 5% CO_2_ to determine the optimal depletion conditions for HK2 cells: each condition was tested in triplicate.

### 4.10. Experimental Design

The cells were washed with PBS after ATP depletion, and conditioned for 4 h at 37 °C and 5% CO_2_ according to the following experimental groups: (a) dATP: HK2 in DMEM-LG; (b) EV: HK2 in DMEM-LG with 3 × 10^7^ EVs/mL; (c) EV-ATP: HK2 in DMEM-LG with 3 × 10^7^ ATP-EVs/mL; (d) Lp: HK2 in DMEM-LG with 3 × 10^7^ liposomes/mL; (e) Lp-ATP: HK2 in DMEM-LG with 3 × 10^7^ ATP-LPs/mL; (f) ATP: HK2 in DMEM-LG with ATP (0.05 nmol/mL).

Not-ATP-depleted HK2 cells cultured in complete medium were used as the positive control (CTRL+), and not-ATP-depleted HK2 cells in DMEM-LG without FCS were used as the negative control (CTRL-). Each condition was performed in quadruplicate.

### 4.11. Assay of Cell Viability

Cell viability was quantified by counting the number of viable cells, defined as cells that remained adherent to the culture flask and excluded Trypan blue (Gibco). Nonadherent cells were removed by two washes with ice-PBS. Adherent cells were harvested by incubation with 0.05% trypsin−0.53 M EDTA ⋅ 4 Na for 10 min at 37 °C (Merck). Trypsin was neutralized by the addition of DMEM containing 10% FCS. Cells were centrifuged for 10 min at 150× *g* and resuspended in DMEM. Viable cell counts were determined by Trypan blue uptake using 0.2% Trypan blue (Gibco) and the cell viability was expressed as the percentage of viable cells relative to the total number of cells.

### 4.12. ATP Quantification in HK2

To avoid interferences in the enzyme-linked immunosorbent assay, HK2 cells of each study group were deproteinized and then incubated with the ATP probe. The results were normalized for 100,000 cells and expressed as nanomoles (nM) in 100 μL or as fold changes.

### 4.13. RNA Extraction and Quantitative Real-Time Polymerase Chain Reaction

Expression levels of apoptotic genes were analyzed on different samples by quantitative real-time PCR (RT- qPCR). Total RNA derived from each sample was extracted and isolated using 300 μL of lysis buffer (TRIzol Reagent, Thermo-Fisher Scientific). Total RNA extraction was performed by using directzol RNA miniprep’s reagents following the manufacturer’s protocol (Zymo Research, Tustin, CA, USA). Total RNA was then quantified by a NanoDropTM (Thermo-Fisher Scientific). cDNAs obtained from 500 ng of RNA were reverse transcribed using an iScriptTM cDNA synthesis kit (Bio-Rad, Hercules, CA, USA) and quantitative PCR analysis was performed using the oligonucleotide primers displayed in Table 1. The reaction was carried out by using a mini-opticon Real-Time PCR system (BioRad, Hercules, CA, USA) and data analysis was performed by CFX Manager software v1.5.534.0511. Gene expressions were analyzed in triplicate and normalized to the glyceraldehyde 3-phosphate dehydrogenase (PGK) gene expression.

### 4.14. Statistical Analysis

Continuous variables were described as mean, range, and standard deviation if they were normally distributed, and as median and interquartile range (p25–p75) if their distribution was skewed. Statistical analyses were carried out using the ordinary one-way analysis of variance test, the Friedman test, paired and unpaired T tests, and the multiple paired T test. Statistical significance was set at *p* < 0.05. Data were analyzed using the GraphPad Prism 5.0 demo program.

## 5. Conclusions

This study provides strong evidence that both extracellular vesicles (EVs) and liposomes can be effectively loaded with ATP and utilized as intracellular energy delivery systems. Our findings show that both liposomes and EVs are efficient in ATP encapsulation, but ATP-loaded EVs are particularly effective at transferring ATP into ischemic HK2 cells, enhancing cell viability and reducing apoptosis, thereby demonstrating their therapeutic potential for ischemic injuries.

However, several challenges remain. Further in vivo studies are needed to confirm these results and evaluate the pharmacokinetics and pharmacodynamics of ATP-loaded EVs under physiological conditions. Additionally, optimizing the homogeneity of EVs and improving ATP loading efficiency will be crucial for maximizing their therapeutic effectiveness.

To our knowledge, this is the first study to demonstrate the direct use of ATP-engineered EVs as an intracellular energy source for ischemic injuries. If successfully translated into clinical practice, this approach could provide substantial benefits, not only in transplantation but also in various ischemic-related conditions. Ultimately, it has the potential to improve patient outcomes by preserving organ function and maintaining cellular energy homeostasis.

## Figures and Tables

**Table 1:** Oligonucleotides primers of apoptosis genes.

BCL-2 BAX	FW TGGAGCTGCAGAGGATGATTG
BCL-2 BAX	RW GGCCTTGAGCACCAGTTTG

## Data Availability

The original contributions presented in this study are included in the article/supplementary material. Further inquiries can be directed to the corresponding author(s).

## Supplementary Materials

The following supporting information can be downloaded at: https://www.mdpi.com/article/10.3390/ijms26073424/s1.

## Abbreviations

2-DG	2-Deoxyglucose
ANOVA	Analysis of Variance
ATP	Adenosine Triphosphate
ATP-EVs	ATP-loaded Extracellular Vesicles
ATP-LPs	ATP- loaded Liposomes
BAX	BCL2 Associated X Protein
BCL2	B-Cell Lymphoma 2
BM	Bone Marrow
BM-MSCs	Bone Marrow derived Mesenchymal Stem Cells
cDNA	Complementary DNA
CD	Cluster of Differentiation
CD11b	Cluster of Differentiation 11b
CD29	Cluster of Differentiation 29
CD45	Cluster of Differentiation 45
CD73	Ecto-5′-Nucleotidase
CD90	Cluster of Differentiation 90
CD105	Cluster of Differentiation 105
cP	Centipoise
CTRL	Control Group
CTRL+	human tubular cells in complete medium (Positive Control)
CTRL-	human tubular cells in medium without serum (Negative Control)
dATPs	Deoxyadenosine Triphosphate/ATP depleted human tubular cells
DLS	Dynamic Light Scattering
DMSO	Dimethyl Sulfoxide
DMEM	Dulbecco’s Modified Eagle Medium
F12	Nutrient Mixture F-12
DSPC	1,2-Distearoyl-sn-glycero-3-phosphocholine
DOPS	1,2-Dioleoyl-sn-glycero-3-phospho-L-serine
EDTA	Ethylenediaminetetraacetic Acid
ELISA	Enzyme-Linked Immunosorbent Assay
EM	Electron Microscopy
EV	ATP-depleted human tubular cells conditioned with extracellular vesicles for 4 h
EV-ATP	ATP-depleted human tubular cells conditioned with ATP-loaded Extracellular Vesicles for 4 h
EVs	Extracellular vesicles
FCS	Fetal Calf Serum
HK2	Human Kidney 2 (cell line)
I/R	Ischemia/Reperfusion
IQR	Interquartile Range
LP	liposomes
Lp-ATP	ATP-depleted human tubular cells conditioned for ATP-loaded liposomes for 4 h
MNCs	Mononuclear Cells
MSCs	Mesenchymal Stem Cells
MSC-EVs	Mesenchymal Stromal Cell derived Extracellular Vesicles
NADH	Nicotinamide Adenine Dinucleotide (Reduced Form)
NTA	Nanoparticle Tracking Analysis
PBS	Phosphate-Buffered Saline
P4	Passage 4
PCR	Polymerase Chain Reaction
PGK	Glyceraldehyde 3-phosphate Dehydrogenase
RC	Regenerated Cellulose
RNA	Ribonucleic Acid
RT-PCR	Real-Time Polymerase Chain Reaction
SHM	Staggered Herringbone Micromixer
TEM	Transmission Electron Microscopy
TFR	Total Flow Rate
TRIzol	Tri Reagent for RNA Extraction
UV	Ultraviolet
Z-potential	Zeta Potential
