# Supplementary material for: Engineered ATP-Loaded Extracellular Vesicles Derived from Mesenchymal Stromal Cells: A Novel Strategy to Counteract Cell ATP Depletion in an In Vitro Model
Source: Int J Mol Sci. 2025 Apr 5;26(7):3424. doi: 10.3390/ijms26073424 (PMC11990007; doi:10.3390/ijms26073424)
Supplement: Supplementary file 1 [file ijms-26-03424-s001.zip › ijms-3536519-supplementary.pdf]

Engineered ATP-Loaded Extracellular Vesicles Derived from Mesenchymal Stromal Cells: A Novel Strategy to Counteract Cell ATP Depletion in an In Vitro Model

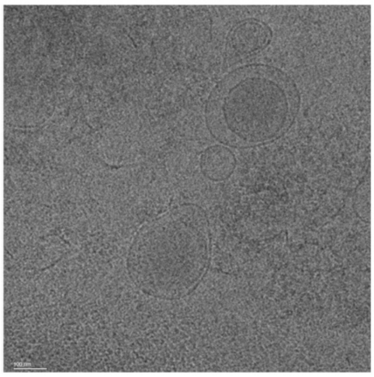

Suppl. Figure S1: Cryo-electron microscopy (Cryo-EM) image of extracellular vesicles (EVs) derived from porcine bone marrow mesenchymal stem cells (MSCs).

The image reveals the characteristic spherical morphology of EVs, with a well-defined lipid bilayer structure, indicating their integrity and stability. The vesicles appear within the expected size range, supporting their classification as exosomes or microvesicles. Cryo-EM provides high-resolution visualization, allowing detailed structural analysis of the EVs, confirming their nanoscale dimensions and membrane organization, which are essential for their biological functionality and therapeutic potential.

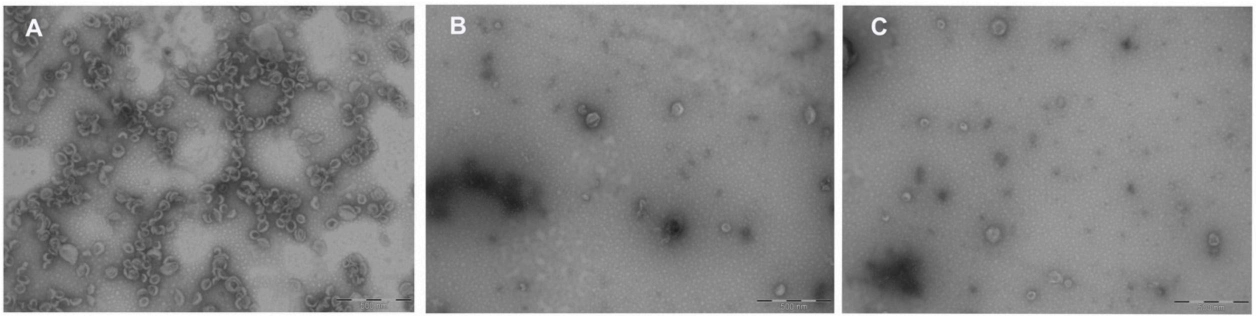

Suppl. Figure S2: TEM images of A) placebo liposomes (DSPC:Chol:DOPS); B) Naïve EVs derived from porcine bone marrow MSCs C) EV after ATP loading. (50K, 150K and 300K magnification).

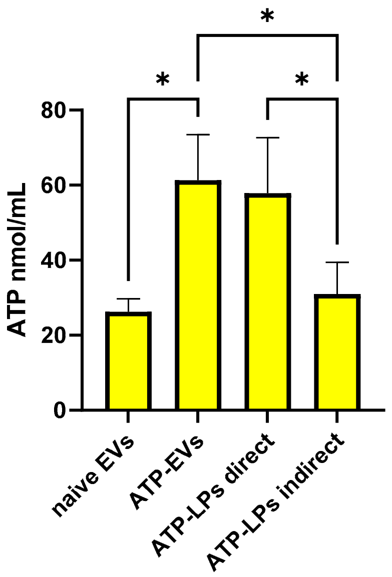

Supplementary Figure S3: ATP level in naïve EVs (EVs), ATP loaded EVs (ATP-EVs) and ATP loaded liposomes using direct (ATP-LPs direct) or indirect (ATP-LPs indirect) methods. Data are expressed as mean and standard deviations. * p<0.05.
